# Facial Neuralgia

**Published:** 1865-07

**Authors:** 


Facial Neuralgia.—An interesting case of this terrible affliction came under my notice, a few weeks back, in a patient sent to me, in consultation, by Dr. Thos. G. Morton, Surgeon to the Pennsylvania Hospital. The patient, who is aged about sixty, has suffered for the past twenty years the most excruciating pain in the right side of the face, generally commencing in the region opposite to the *infra-orbital* foramen, and radiating from this point to the various parts to which the nerves passing from the foramen are distributed, viz., upper lip, alae of the nose, and lower eyelid. The paroxysms, as a general thing, make their appearance with a tickling or pricking sensation of the cheek, which increases in severity until the pain becomes most agonizing and unendurable. During the extended period to which he has been a victim to this fearful malady, recourse has been had to all the medical agents that science has recognized as valuable under such circumstances, but they have proved of no advantage to him. A short time since all the teeth in the right superior maxillæ were extracted, under the impression that their removal would prove of advantage; the only result obtained, however, was to deprive him of a number of excellent masticating organs (for they were all perfectly sound) without securing any amelioration from his sufferings. It was some months after this that he called upon me. After listening to the description of his sufferings, and making a careful examination of his mouth and face, I informed him that the disease he labored under evidently had its origin in the upper jaw, the pain being due to compression of a branch of the second branch of the fifth pair of nerves in the infra-orbital canal, from osteal or periosteal thickening of the parietes of the canal; and that the only plan by which he could obtain relief was to submit to the trephining of the jaw, so that the portion of compressed nerve could be removed. In a subsequent interview with Dr. Morton, I found he had formed the same opinion as that expressed above, and proposes to perform the operation at the earliest moment possible. The success which Carnochan has met with, in the three cases described by him, fully justifies the performance of such an operation.

No one can have a proper conception of the terrible character of this affliction, unless he has witnessed the manifestations of suffering, on the part of a patient laboring under an attack; then the writhing form, the rolling eyes, the streaming tears, the quivering features, the constant cries of anguish, prove that the pain is fearful in the extreme, and very, very hard to endure.

The most forcible description that has ever been presented of the sufferings of a victim to this terrible scourge is contained in the following letter, taken from Carnachan's *Contributions to Operative Surgery and Surgical Pathology.*

"In the year 1811, a letter appeared in the *Philadelphia Medical Museum,* written in 1807, by Dr. Jones, an eminent physician of that time in the City of New York, and addressed by him to the celebrated Dr. Benjamin Rush. This letter is a statement of Dr. Jones' own case, he having been, for many years, a victim to intense neuralgia of the face. The narrative is drawn up with much clearness of expression, and is worthy of insertion here, as emanating from a highly intelligent source, and as presenting a most graphic description of the excruciating and fearful agony experienced by those who are afflicted with the intense form of this disease."

Dr. Jones' letter runs thus:—

"New York, Oct. 15th, 1807.

"About the middle of July, 1806, I began to feel an uneasy sensation in the gum of the upper jaw, on the right side, at a point whence one of the molars, in a loosened state, had a long time before been taken away; the socket of which, however, had already been absorbed or filled up, and the gum or alveolar border of the jaw was even, firm, and apparently sound; yet whenever I washed my face, or moved my hand gently over the cheek, a latent disease was perceptible. It gradually increased, till it became so extremely painful, that at times I was compelled to cry out with the intolerable anguish it occasioned. Eating, drinking, speaking, hawking and spitting, sneezing, coughing, and blowing the nose, would either of them, in a moment, awaken the most poignant and acute pain. Even touching the eye with the finger, slightly rubbing the forehead, putting on a pair of spectacles, or only opening the mouth wide, would excite a return of the pain. Taking into the mouth anything hot, cold, or acid, was sure to produce the effect with aggravated violence. The necessary mastication forbade the use, in a great measure, of solid food. Combing the hair, shaving the right corner of the mouth, reading aloud, or anything that gave the slightest motion to the muscles of the face, would occasion in the part a throbbing, which seemed to begin like the vibration of a musical cord, extending its effects to the cheek, the eye, the nose, up to the scalp on that side of the head, and, after continuing for a few seconds, sometimes a few minutes, and latterly for fifteen or twenty minutes, it would cease, and the part which had been affected would then feel as well as if nothing had happened. The pain at length began to be felt severely, upon touching the left eye, yet, whatever it was that excited the pain, it always centered in the gum, on the right side of the upper jaw.

"It continued in this way, except increasing in the duration of each paroxysm, till August 28th, 1807, when it almost, or entirely left me, and I began to flatter myself it had been vanquished by the use of hemlock; but in two or three days it returned, and became as violent and excruciating as ever. The gum where the disease is seated, I have already mentioned, is to all appearance perfectly well, sound, and smooth, with no visible vestige of disease about it; and there had been no tooth in the spot for some considerable time before the disease commenced. The gum has been repeatedly divided by numerous longitudinal and transverse incisions down to the bone. This operation in the beginning of the complaint, never failed to assuage the pain for a time; but the relief seldom lasted till the gum had healed. Electricity was tried for a long time, to no purpose. A number of topical applications were made; the part was covered with a blister externally, and various embrocations applied to the cheek without advantage. Opium was taken in large quantities, and the tincture applied to the part, both externally and internally. Warm and cold bathing were alternately applied to the head and face. The latter application occasioned the most exquisite misery. Peruvian bark in substance was taken in large doses. Huxham's tincture of the bark, with wine, was used very liberally. The volatile alkali, in a decoction of bark, was taken agreeably to Dr. Fothergill's prescription, in a recent publication of the *successful* treatment of a case of this very formidable disease. None of these things effected any change that promised the final removal of the complaint. I then began a steady use of the extract of hemlock, taking one grain night and morning, till I had risen to the quantity of fourteen grains every four hours. This was continued till September 22d, 1807, when, finding it produced no diminution of the pain, I omitted the use of it for one week, when, the pain recurring more frequently, as well as more severely than before, I again had recourse to the hemlock, and am now, without much benefit from it, or very sanguine hopes in its curative efficacy, still persevering in the daily use of it. I have been advised to have the actual cautery applied to the gum; but am apprehensive that so harsh and violent an application would bring on tetanus with all its alarming and distressful consequences, and have therefore concluded not to adventure upon the experiment.

"This complaint will be admitted, I presume, to be a nervous affection; but what remains mysterious, and seems to ask for explanation is, that if a local disease, cutting the gum all to pieces, dissecting and dissevering it in all directions, should not, by dividing the nerves and insulating them from all connection with the sensorium, destroy their sensibility, or at least their power of *communicating* pain. I am aware that while their continuity is preserved, denudating the nerves, so as to expose them to the contact of the common air, does often cause them to assume a morbid action productive of extreme pain; but this did not happen in the present instance, for, when the scarifications did least good, they did not seem, in the smallest degree to increase the evil.

"I am upwards of sixty-two years of age, and, except this complaint, my general health and appetite are good; I have, indeed, a small ulcer in one of my ankles, which has been healed, but from irritation has broken out again, and at present does not seem much disposed to cicatrize. I have, during my whole life, been in the habit of using steady exercise, and have lived as regularly and as temperately as any man.

"If, sir, on considering this case, which has thus far eluded the best professional skill of this city, anything occurs to your mind, which would throw light on the nature and character of my complaint, and especially if your extensive research and experience should have furnished you with anything promising for the treatment of it, by communicating your opinions and prescriptions, you will not only give to the world a new proof of your pre-eminent skill, but you will at the same time subserve the interests of humanity, by administering comfort and relief to an old man, suffering under the most afflictive distress; you will cause his winter sun, now dimmed and clouded with despondency, to shine out afresh with a mild and cheering ray, and at last go down in a serene sky; and, what may be of still more value in your estimation, you will enjoy the conscious merit of acting in conformity with the benign precepts and bright example of the *Great Physician*, who, while on earth, delighted in relieving the distresses of his brethren."

"Dr. Rush replied to this letter, recommending such remedies as he supposed might be most effective in arresting the fearful disease. We learn, however, from the following extract, taken from another letter to Dr. Rush, written two years after the one just quoted, that all the remedies which were used had proved unavailing. Dr Jones, in his second letter, dated December 9th, 18 9, thus alludes to his condition at the time. ----- 'My disease, sir, still maintains its place, unmoved by all that weight, and force, and complication of machinery which medical science and skill have devised and applied. The most promising and judicious prescriptions, derived either from enlightened reasoning, or from experience, or from learned research, have alike failed, in this baffling disease, to answer the expectations reasonably entertained of them.'

"I am informed by a venerable and esteemed physician, still living in this city, that Dr. Jones was afterward subjected to the operation of division of the infra-orbital nerve on the cheek, but without receiving any material benefit; and further, that, unrelieved by any of the curative means he had so patiently resorted to, he succumbed und r the prolonged and agonizing suffering which he had endured."

